# β-Sitosterol differentially regulates key metabolites for growth improvement and stress tolerance in rice plants during prolonged UV-B stress

**DOI:** 10.1186/s43141-021-00183-6

**Published:** 2021-05-29

**Authors:** Raheel Shahzad, Mohamed Ewas, Putri Widyanti Harlina, Shahid Ullah Khan, Pan Zhenyuan, Xinhui Nie, Elsayed Nishawy

**Affiliations:** 1 Department of Biotechnology, Faculty of Science and Technology, Universitas Muhammadiyah Bandung, Bandung, West Java 40614 Indonesia; 2 National Key Laboratory of Crop Genetic Improvement, Huazhong Agricultural University, Wuhan, 430070 China; 3 Department of Plant Genetic Resources, Desert Research Center, Cairo, 11753 Egypt; 4 Department of Food Technology, Faculty of Science and Technology, Universitas Muhammadiyah Bandung, Bandung, West Java 40614 Indonesia; 5 Key Laboratory of Oasis Ecology Agricultural of Xinjiang Bingtuan, Agricultural College, Shihezi University, Shihezi, Xinjiang, 832003 China

**Keywords:** Rice, UV-B stress, β-Sitosterol, Physiological response, Metabolic analysis

## Abstract

**Background:**

Elevated ultraviolet-B (UV-B) radiation is potentially deleterious to many organisms specifically crop plants and has become a global challenge. Rice is an exceptionally important staple food which is grown worldwide, and many efforts have been done recently to improve rice varieties against UV-B stress. This current study aims to investigate the effects of exogenous application of β-sitosterol (βSito) on growth improvement and tolerance level of rice plants against prolonged UV-B stress. The physiological and metabolic responses were evaluated in rice plants not supplemented with βSito (Nβ) and those supplemented with βSito (Sβ).

**Results:**

The Nβ and Sβ plants were grown under non-stress (ns) and under prolonged UV-B stress (uvs) conditions and termed as Nβ^ns^, Sβ^ns^ and Nβ^uvs^, Sβ^uvs^, respectively. The application of βSito contributes positively under non-stress and specifically to UV-B stress in terms of improving numerous physiological parameters associated with growth and development such as shoot and root length, RWC, whole plant biomass, chlorophyll pigments, and photosynthetic-related parameters (Pn, Gs, Tr, WUEi, Fv/Fm, and NPQ) in Sβ compared with Nβ plants. Moreover, enhanced oxidative stress tolerance of Sβ^uvs^ vs. Nβ^uvs^ plants under stress was attributed to low levels of ROS and substantial trigger in activities of antioxidant enzymes (SOD, POD, CAT, and APX). Metabolic analysis was performed using GC-TOFMS, which revealed higher accumulation of several key metabolites including organic acids, sugars, amino acids, and others in Sβ^uvs^ vs. Nβ^uvs^ plants, which were mainly reduced in Nβ plants under stress vs. non-stress conditions.

**Conclusion:**

These results provide useful data regarding the important role of βSito on growth maintenance and modulation of several metabolites associated with osmotic and redox adjustments during UV-B stress tolerance in rice plants. Importantly, βSito-regulated plasticity could further be explored specifically in relation to different environmental stresses in other economically useful crop plants.

**Supplementary Information:**

The online version contains supplementary material available at 10.1186/s43141-021-00183-6.

## Background

Plants need continuous sunlight for plant growth and development. However, ultraviolet (UV) radiation in natural sunlight is unavoidable. The increasing evidence in which stratospheric ozone is influenced by climate change and thus UV radiation received by earth’s surface eventually lead to changes in agricultural ecosystems and significantly influence plant yield and crop productivity [1]. It is widely recognized that ultraviolet B (UV-B) radiation generally exerts a negative impact on most agricultural crops and plants in number of different ways. For instance, exposure of plants to UV-B radiation negatively affects cell proliferation and expansion, hypocotyl growth, and leaf and seedling growth [2–4]. The most damaging effect of UV-B radiation on plants is inhibiting the photosynthetic processes which subsequently could affect plant biomass. Previous studies have shown that high UV-B irradiation can severely disrupt PSII reaction center proteins, damage electron transport chain, and thus minimize the plant growth [5]. Other indirect effects of UV-B radiation have also been reported such as changes in photosynthetic and non-photosynthetic pigments, stomatal conductance, and stress-related hormones which cause irreversible injuries to plant growth and survival [6].

Like most other stress factors, UV-B irradiation markedly induces an oxidative burst produced by increased cellular levels of reactive oxygen species (ROS) and therefore cause perpetual oxidative damages to plants [7]. In plants, ROS exist in ionic and/or molecular states. Ionic states include hydroxyl radicals (•OH) and superoxide anions (O_2_·−), while molecular states mainly include hydrogen peroxide (H_2_O_2_) and singlet oxygen (O_2_). Generally, ROS perform dual function depending on the given stress situation, and not only cause cell death but also function as a signaling molecule in response to stress. Additionally, ROS control epigenetic events and the level of phytohormones to execute developmental processes and stress responses in plants [8]. However, normally, it is extremely difficult to distinguish between the cytotoxic and signaling events that are induced by a particular ROS. As a natural defense mechanism in plants and under normal conditions, excessive ROS levels can be efficiently scavenged via antioxidant enzymes [9]. Several plant antioxidant enzymes such as catalase (CAT), superoxide dismutase (SOD), peroxidase (POD), glutathione peroxidase (GPx), ascorbate peroxidase (APx), and others play critical roles in removing reactive oxygen species in cells directly or indirectly, thus ensuring normal metabolic reactions against the toxic effect of ROS generated by UV-B stress [10].

Interestingly, plants accumulate several metabolites (primary and secondary metabolites) in order to protect the plants from adverse conditions caused by various abiotic stresses including UV-B irradiation [11, 12]. In higher plants, a wide variety of secondary metabolites (alkaloids, terpenoids, flavonoids, etc.) are synthesized from primary metabolites (such as sugars, lipids, and amino acids). In plants, differential accumulation of a variety of metabolites is not directly related to their central energy metabolism or structural integrity. In fact, plant metabolites and their distribution and diversity are reflective of their essential functions in plant stress response. Furthermore, the increase of different metabolites in stressed plants is a non-enzymatic mechanism to enhance antioxidant capability in plants [13]. Generally, these diverse solutes exhibit a high level of solubility in the cellular milieu, and their accumulation does not affect enzyme activities even at high concentrations. Moreover, the chemical nature of these osmoprotectants is very diverse that includes amino acids (proline, leucine, iso-leucine, tryptophan), amines, and γ-amino-*N*-butyric acid (GABA). Furthermore, carbohydrates (glucose, sucrose, fructose) and polyols (inositol, myo-inositol), as well as different organic acids (oxalic acid, salicylic acid, ferulaic acid, etc.), accumulate in response to osmotic stress in plants [14].

The β-sitosterol is an active phytosterol that exists in most plant species and significantly influences membrane fluidity and permeability. In general, plant sterols or phytosterols serve as integral components of the membrane lipid bilayer, and they play various functions in plants, ranging from regulation of growth and development to stress resistance [13, 15]. With regard to β-sitosterol (βSito) and its role in stress tolerance mechanisms, recently, a number of studies show that βSito could effectively regulate several biological events to enhance plant resistance against stress factors such as water stress [16] and salt stress [17], as well as role in plant-pathogen interaction [18]. Nevertheless, it is important to mention that most of these studies still lack the knowledge to fully understand the underlying regulatory mechanisms. More efforts regarding βSito and its regulatory functions in mitigating stress are therefore needed to comprehend its critical role for plant protection.

Rice is an exceptionally important staple food which is grown worldwide due to its high demand and economic value. Given the fact that UV-B stress can negatively affect numerous morpho-physiological events and metabolic profile in different plant species as reported in earlier studies [3, 19]. Notably, rice cultivars also show extreme sensitivity to UV-B light [20], and thus, more efforts are required in finding ways to improve resistance and maintain high productivity of rice. Recent efforts to enhance rice tolerance for UV-B stress are largely associated with transgenic approaches [12, 21, 22]. Nonetheless, the development of transgenic plants is a long-term and expensive process. On the other hand, the exogenous application of phytosterols in research for improving abiotic stress tolerance in rice holds many advantages such as higher effectiveness, low cost of handling, and its environmentally friendly attribute. Considering all these points in mind, we attempt to explore the effects of supplementation of βSito on metabolic and physiological processes in rice plants subjected to prolonged UV-B stress. To the best of our knowledge, such study is conducted for the first time to reveal the molecular mechanisms of UV-B stress tolerance conferred through exogenous application of βSito in rice plants. Therefore, our specific goals for conducting this research were (1) whether βSito could really enhance tolerance of rice plants under prolonged UV-B irradiation and (2) to evaluate oxidative damages in βSito treated and untreated plants, further (3) to investigate and compare physiological parameters and metabolite changes in rice plants with or without βSito both under non-stress and UV-B stress.

## Methods

### Plant material, growth conditions, and UV treatment

In this study, seeds of rice plant (*Oryza sativa* L. spp. japonica) were obtained from cultivar Zhonghua 11 (ZH11), which is commonly grown japonica rice in China. Seeds were first surface-disinfected with 5% NaClO for 10–12 min and washed several times with distilled water until clean. The seeds were soaked in sterile water for about 48 h in a 28 °C incubator, and the water was removed. Next, we placed healthy seeds with uniform size in sterilized Petri dishes, and this culture was incubated in a biochemical incubator for 24 h at 28 °C for pre-germination. After this, the seeds were allowed to germinate normally under photosynthetically active radiation (PPFD of 350 μmol quanta m^−2^ s^−1^) in a growth chamber with a light/dark cycle of 14/10 h for about 1 week. We selected uniformly grown seedlings and transferred them to ½ Hoagland nutrients solution and let them grow for one more week under the same growth conditions. We purchased commercially available β-sitosterol (βSito; S1270, synthetic, ≥ 95%, Sigma Aldrich). βSito was prepared at a concentration of 150 μmol L^− 1^ β-sitosterol solution dissolved in a small amount of 100% ethanol and adjusted to the required concentration with nutrient solution. Rice plants were distributed into two groups, βSito-treated plants (Sβ) and βSito-untreated plants (Nβ). For βSito treatment (root feeding), we mixed βSito solution into nutrient solution, and the culture was grown for about 20 days. For untreated plants, we grow the culture in nutrient solution without βSito solution.

The Nβ and Sβ plants were grown in non-stress (ns) and under prolonged UV-B stress (uvs) conditions, and thus, we named the plants under non-stress conditions as Nβ^ns^ and Sβ^ns^, and those subjected to prolonged UV-B stress were termed as Nβ^uvs^ and Sβ^uvs^ plants, respectively. For prolonged UV-B stress treatment in Nβ and Sβ plants, we provided UV-B radiation (315 ± 20 nm) through UV-B lamps (UV-B313EL, Beijing Lighting Research Institute, Beijing, China) and plastic filters. UV-B fluorescent lamps were erected at a certain height above the rice seedlings for constant exposure to UV light. Rice seedlings (about 35 days old) were subjected to UV-B radiation each day for 6 h between 10:00 a.m. and 4:00 p.m. continuously for 5 days following a previous study by others [23]. For non-stress conditions, we grow Nβ and Sβ plants under normal light conditions. Leaf samples were collected and stored from each group of plants (UV-B treated and untreated) in liquid nitrogen and stored at −80 °C for further analysis.

### Analysis of growth and morphological characters

Twenty seedlings were selected randomly from plant containers and used for the determination of growth and morphological characters after UV-B treatment. After harvesting the plants, we measured the shoot and root length by using a standard metallic ruler. To evaluate the biomass differences, we sampled five plants from each container, and fresh and dry weight of twenty individual plants from each group (non-stress and UV stress) was measured by using the electronic analytical balance (BSA224S, Sartorius, Taiwan). For calculating relative leaf water content (RWC, %) in each group of plants under non-stress and UV stress conditions, we used the following equation:

$$ \mathrm{Relative}\ \mathrm{water}\ \mathrm{content}\ \left(\%\right)=\left(\left(\mathrm{Fresh}\ \mathrm{weight}--\mathrm{Dry}\ \mathrm{weight}\right)/\mathrm{Fresh}\ \mathrm{weight}\right)\times 100 $$

### Analysis of photosynthetic-related physiological parameters

We measured photosynthetic-related pigments including chlorophyll a (Chl a), chlorophyll b (Chl b), total chlorophyll, and carotenoids (Caro). The leaf samples from non-stress as well as UV-B-treated plants were crushed using liquid nitrogen, and extraction of pigments was carried out by the dimethyl sulfoxide (DMSO) method. The extraction sample was prepared (100 mg of fresh sample) in dark at the room temperature in acetone (80%), and absorbance readings were then determined with a UV/VIS spectrophotometer (Shimadzu UV-160, Kyoto, Japan). The chlorophyll content of each leaf was based on the average of three readings. The measurement was completed using two biological replicates (taken from the same container), with six plants per replicate.

For photosynthetic-related gas exchange parameters, fully developed leaf from the middle of each seedling were selected from Nβ and Sβ plants (non-stress and UV stress), and measurements were recorded between 8.00 and 10:00 a.m. by a Li-6400 Portable Photosynthesis System (Li-Cor Inc., Lincoln, NE, USA). To achieve full photosynthetic induction, the samples were illuminated first with saturated photosynthetic photon flux density (PPFD) provided by light source (LED) for 30 min before measurements. Measurements of net photosynthetic rate (Pn), rate of transpiration (Tr), and stomatal conductance (Gs) were recorded simultaneously as described previously [24]. The calculation of intrinsic water use efficiency (WUEi) was based on the ratio of Pn and Tr.

Chlorophyll fluorescence parameters were measured on fully expanded leaves of the same plants that were selected for gas exchange analysis, and data were analyzed in non-stress and UV-B-treated samples by using LICOR 6400 system. The fully expanded leaves were dark acclimated in the LI-6400XT leaf chamber for 30 min at 28 °C prior to measuring the minimum fluorescence (Fo) and maximum fluorescence (Fm). The maximum quantum yield of PSII (Fv/Fm) was calculated as (Fm − Fo)/Fm according to an earlier study [25]. The non-photochemical quenching (NPQ) coefficient was determined according to previous study [26].

### Determination of reactive oxygen species and MDA contents

Leaf samples from non-stress and UV-treated plants were selected, and the concentration of H_2_O_2_ was prepared and estimated following the method [27]. The absorbance of the reaction was recorded at 390 nm, and the content of H_2_O_2_ was calculated based on a standard curve. To determine O_2_ content, the assay was performed as described earlier [28]. The absorption of the reaction mixture was recorded at 530 nm. O_2_ was calculated according to a standard curve based on sodium nitrite. To examine superoxide anion radicals in leaf samples from Nβ and Sβ plants, we first cut the leaves into small sections and immersed them into 40 mL staining solution (0.1% w/v) NBT including 10 mM sodium azide and 50 mM potassium phosphate as mentioned in previous study [29]. The photographs of the leaf samples were then captured after 24 h of staining showing the presence (blue dots) or absence (white clear background) of radical ions.

### Determination of antioxidant enzyme activities

Activities of antioxidant enzymes were determined from the leaf samples of Nβ and Sβ plants (non-stress and UV stress). The extracts for SOD, POD, and CAT activities were prepared by weighing about 0.2 g of leaf samples, which were freezed using liquid nitrogen to avoid proteolytic activity. The samples were grounded with 5 mL extraction buffer (0.1 M phosphate buffer, pH 7.5, containing 0.5 mM EDTA), followed by centrifugation for 20 min at 15,000*g* at chill temperature (4 °C), and the supernatant was collected and assayed for enzyme activity as described in previous study [30]. For APX activity, 300 μL crude leaf extract was incubated at 28 °C with 600 μL 0.05 M potassium phosphate buffer (pH 6), 0.5 mM ascorbate, and 100 μL 2 mM H_2_O_2_. APX activity was analyzed as a decrease in absorbance during 5 min at 290 nm as described in previous method [31]. Each experiment was performed in triplicate.

### Metabolite extraction, analysis, and quantification based on GC-TOFMS

We grow Nβ and Sβ plants under non-stress and UV-B stress conditions, and samples from about 40-day-old rice seedlings were harvested from Nβ and Sβ plants both under non-stress and stress conditions and stored at −80 °C after freezing in liquid nitrogen. Metabolites were extracted from the leaf tissue (~ 100 mg) and derivatized as described earlier [32]. A sample volume of 1 μL was then injected, and samples were analyzed using comprehensive two-dimensional gas chromatography/time-of-fight mass spectrometry (GC-TOFMS, Pegasus 4D, LECO Corporation, St. Joseph, MI, USA). Peak detection and mass spectra deconvolution were performed with the Leco Chroma-TOF software v.2.25. The resulting files were processed using metabolomics database. Metabolite data was normalized using ribitol (internal standard). Peak assignment of each metabolite was confirmed by comparison with a reference mass spectral library from the National Institute of Standards and Technology (NIST, Gaithersburg, MD, USA) and the public domain mass spectral library of the Max Planck Institute for Plant Physiology, Golm, Germany (http://csbdb.mpimp-golm.mpg.de/). Relative levels of selected metabolites were examined by automatic integration of the peak areas of selective ions separated from the total ion chromatogram.

### Statistical analysis

The analysis of variance (ANOVA) was performed using the SAS statistical software (SAS 9.1, SAS Institute, Cary, NC, USA) for all measured parameters in this study. Significant differences of the means between non-stress and stress treatments were evaluated using Student’s t test. Experiments were repeated three times with similar results.

## Results

### Effects of β-sitosterol on plant growth and water status of rice seedlings exposed to prolonged UV-B stress

**Fig. 1:**
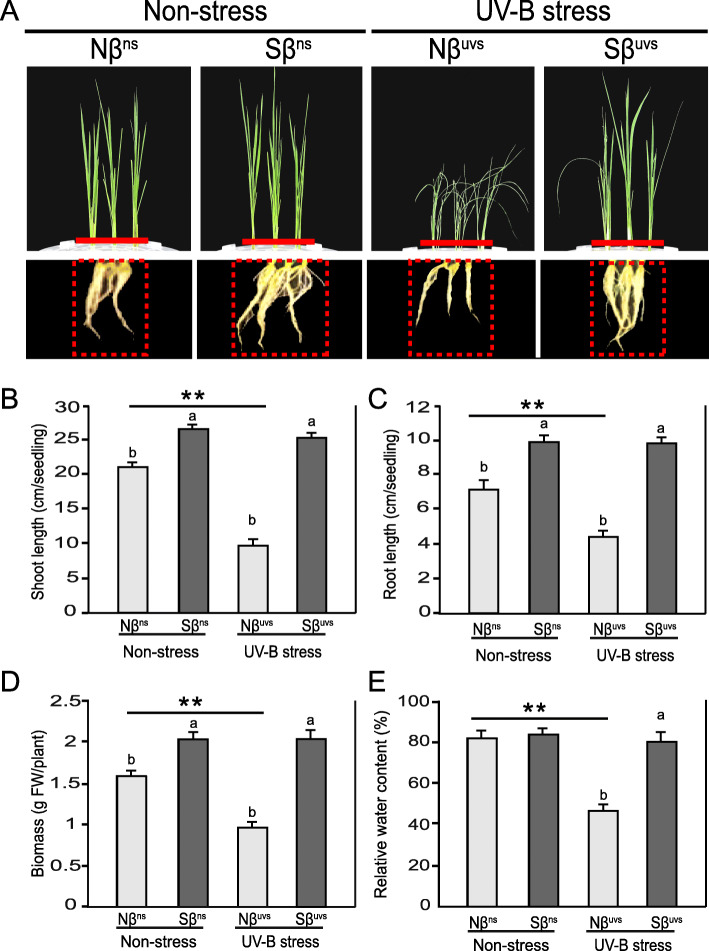
Effects of prolonged UV-B stress on Nβ and Sβ seedlings. **A** Photographs of the shoots (upper panel) and roots (lower panel) of Nβ and Sβ plants under non-stress and after 5-day-long (6 h/day) UV-B stress. **B** Shoot length, **C** root length, **D** plant biomass represented in g FW/plant, and **E** relative water contents from the leaves of Nβ and Sβ seedlings under non-stress and stress conditions were subsequently measured for growth performance. The means with different letters are significantly different (P < 0.05) within the same treatment (Nβ^ns^ vs. Sβ^ns^ and Nβ^uvs^ vs. Sβ^uvs^). Lines with asterisks indicate significant differences between non-stress and UV-B stress conditions of the same plants (Nβ^ns^ vs. Nβ^uvs^ and Sβ^ns^ vs. Sβ^uvs^), as determined by Student’s t test (*P < 0.05, **P < 0.01)

Plants are vulnerable to absorption of strong UV-B light, and this can affect different aspects of plant growth and development. According to the objectives of this study, rice plants not supplemented with βSito (Nβ) were grown in normal light under non-stress (ns) and under prolonged UV-B stress (uvs) conditions (termed as Nβ^ns^ and Nβ^uvs^, respectively). Similarly, rice plants supplemented with βSito (Sβ) were grown under non-stress and UV-B stress conditions (and termed as Sβ^ns^ and Sβ^uvs^, respectively) for comparative analysis. Initially, we have observed phenotypic changes which clearly show markedly enhanced tolerance of Sβ^uvs^ plants; however, Nβ^uvs^ plants were severely damaged, and all the leaves become twisted and crinkled under stress conditions (Fig. 1A). Under non-stress conditions, shoot and root lengths of Sβ plants were increased as compared to Nβ plants (Sβ^ns^ vs. Nβ^ns^). Upon prolonged exposure to UV-B stress, the shoot and root length was not affected in Sβ^uvs^ plants (Sβ^uvs^ vs. Sβ^ns^); in contrast, a significant decrease in shoot (57.15% decrease in Nβ^uvs^ vs. Nβ^ns^, 64% decrease in Nβ^uvs^ vs. Sβ^uvs^) and root (40% decrease in Nβ^uvs^ vs. Nβ^ns^, 42% decrease in Nβ^uvs^ vs. Sβ^uvs^) length was noticed in Nβ^uvs^ plants (Fig. 1B, C). Another aspect of measuring plant growth under stress conditions is the evaluation of whole plant biomass. Under normal light conditions (non-stress), our results demonstrate that plant biomass for Sβ plants was increased up to 33% than Nβ plants (Sβ^ns^ vs. Nβ^ns^). Notably, the plant biomass of Sβ^uvs^ plants was not affected even after UV-B stress. Contrastingly, we found a substantial decrease of plant biomass in Nβ^uvs^ plants, i.e., nearly 52% lower as compared with Sβ^uvs^ plants under prolonged UV-B irradiation stress (Fig. 1D). To check the water status of plants, we analyzed leaf relative water content both in Nβ and Sβ plants under non-stress and UV-B stress. No significant difference was observed between Nβ^ns^ and Sβ^ns^ plants under normal conditions, but when plants were subjected to prolonged UV-B stress, a marked decrease of 44% was noticed in Nβ^uvs^ plants as compared to Sβ^uvs^ plants (Fig. 1E). Overall, our result reveals the positive effect of βSito on the growth performance and water status of rice plants under prolonged UV-B stress conditions.

### Supplementation of β-sitosterol affects photosynthetic pigments and ultrastructure of chloroplast in rice plants exposed to prolonged UV-B irradiation

**Fig. 2:**
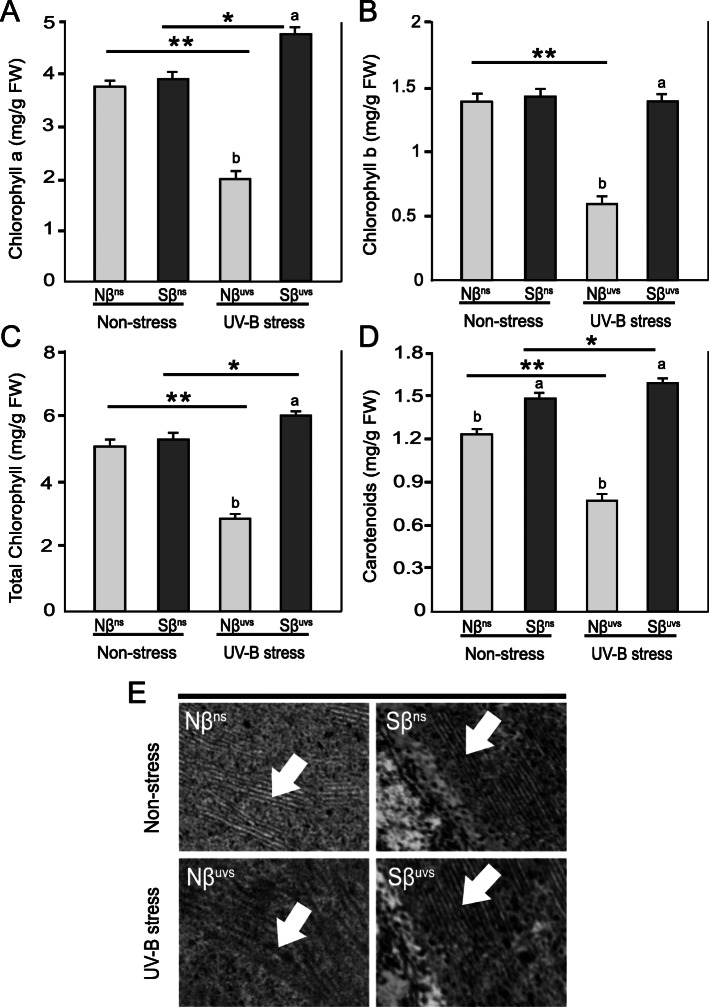
Effects of prolonged UV-B stress on photosynthesis-related pigments and ultrastructure of chloroplast in Nβ and Sβ seedlings. Measurement of pigment contents: **A** Chlorophyll a (Chl a), **B** chlorophyll b (Chl b), **C** total chlorophyll, and **D** carotenoids (Caro) in Nβ and Sβ plants under non-stress and stress conditions. **E** Ultrastructure of the chloroplasts of Nβ and Sβ plants under non-stress and UV-B stress, showing more disintegration of grana stacks in Nβ under stress conditions. Data are shown as means of three independent experiments and different letters showing significant differences (P < 0.05) within the same treatment (Nβ^ns^ vs. Sβ^ns^ and Nβ^uvs^ vs. Sβ^uvs^). Lines with asterisks indicate significant differences between non-stress and UV-B stress conditions of the same plants (Nβ^ns^ vs. Nβ^uvs^ and Sβ^ns^ vs. Sβ^uvs^), as determined by Student’s t test (*P < 0.05, **P < 0.01)

Photosynthetic pigments Chl a, Chl b, and Caro are important for capturing light to perform efficient photosynthesis and are good indicators for plant adaptability to stress conditions. Therefore, we measured the content of these pigments in Nβ and Sβ plants under non-stress and UV-B stress. Under non-stress conditions, there were no significant differences for all the photosynthetic pigments between Nβ^ns^ and Sβ^ns^ plants except for Caro, contents of which were relatively increased in Sβ^ns^ plants (Fig. 2D). Interestingly, there was a significant reduction in the levels of Chl a, Chl b, total chlorophyll, and Caro in Nβ^uvs^ plants after exposure to UV-B stress (Nβ^uvs^ vs. Nβ^ns^ and Nβ^uvs^ vs. Sβ^uvs^). Conversely, we have found that the contents of Chl a, total chlorophyll, and Caro in Sβ^uvs^ plants were significantly higher (Sβ^uvs^ vs. Sβ^ns^ and Sβ^uvs^ vs. Nβ^uvs^), whereas the content of Chl b was relatively constant (Sβ^uvs^ vs. Sβ^ns^) after stress (Fig. 2A–D). Moreover, we have also examined any effects on the ultrastructure of chloroplast in Nβ and Sβ plants subjected to prolonged UV-B stress. For this purpose, we make use of transmission electron microscopy which shows disorganization of grana stacks in the chloroplasts of leaves from Nβ^uvs^ plants after exposure to prolonged UV-B irradiation, while no significant effect was observed in Sβ^uvs^ as compared to non-stress plants (Fig. 2E). Collectively, the results demonstrated that βSito was involved in maintaining chloroplast development and chlorophyll synthesis under stress conditions thus showing superior performance of Sβ plants compared to Nβ plants.

### Effects of β-sitosterol on net photosynthesis of rice plants exposed to prolonged UV-B stress

**Fig. 3:**
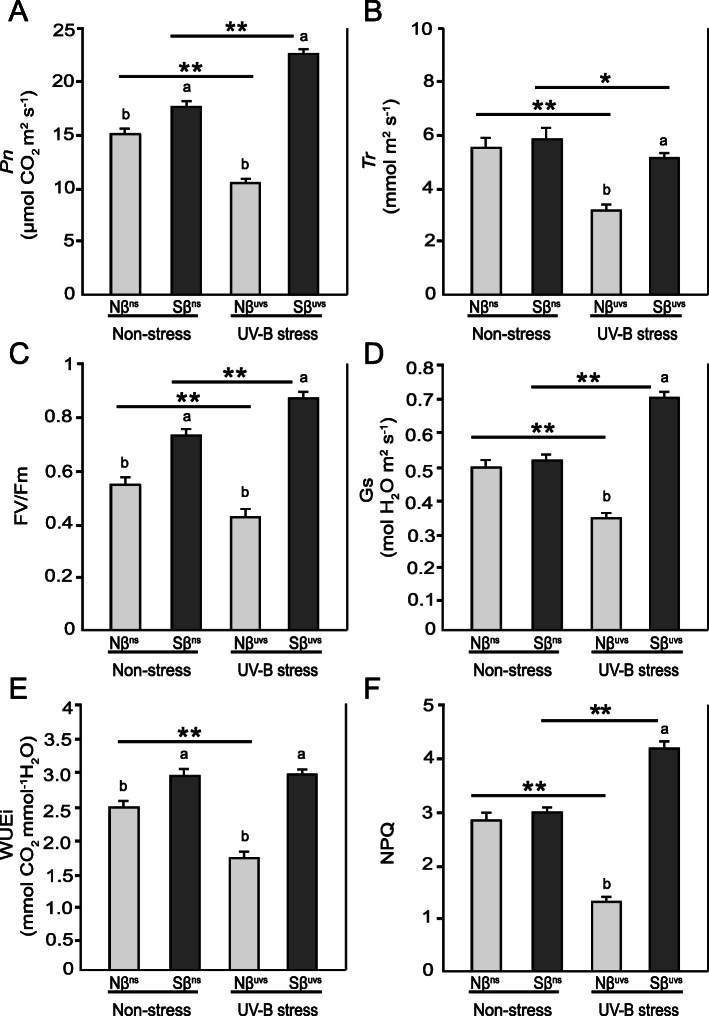
Physiological responses of Nβ and Sβ seedlings under non-stress and stress conditions. **A** The net photosynthetic rate (Pn), **B** transpiration rate (Tr), **C** PSII efficiency (Fv/Fm), **D** stomatal conductance (Gs), **E** water use efficiency (WUEi), and **F** non-photochemical quenching (NPQ) values were recorded for Nβ and Sβ plants under non-stress and stress conditions. Statistical analyses were conducted as described in Fig. 1

Of all the biological processes, photosynthesis is the central and most crucial process for measuring the physiological characteristics of plants under various environmental challenges. Any change in photosynthetic-related parameters affects the overall sensitivity of photosynthesis and subsequently changes the plant physiology. In this study, almost 27.78% increase in Pn was recorded in Sβ^uvs^ (vs. Sβ^ns^) plants exposed to UV-B stress; however, no change was seen in WUEi, but Tr was reduced slightly after stress (Fig. 3). A similar increasing trend was noted for Gs, Fv/Fm, and NPQ in Sβ^uvs^ vs. Sβ^ns^ plants after stress (Fig. 3). Furthermore, under non-stress conditions, Pn, Fv/Fm, and WUEi were also enhanced in Sβ^ns^ vs. Nβ^ns^. In contrast, photosynthesis was negatively affected in Nβ^uvs^ plants, and as we expected, the values for all these parameters were significantly reduced in Nβ^uvs^ plants after UV-B irradiation (Fig. 3). In case of Pn, almost 52.20% and 26.67% reduction (Nβ^uvs^ vs. Sβ^uvs^ and Nβ^uvs^ vs. Nβ^ns^, respectively) was observed in Nβ^uvs^ plants (Fig. 3A). Notably, this result was also consistent with reduced chlorophyll content and a decrease of plant biomass in Nβ^uvs^ plants. In conclusion, these results clearly indicate the positive effects of βSito application on net photosynthesis and associated gas exchange parameters in Sβ plants under prolonged UV-B irradiation.

### Effects of β-sitosterol on oxidative stability of UV-B exposed rice plants

**Fig. 4:**
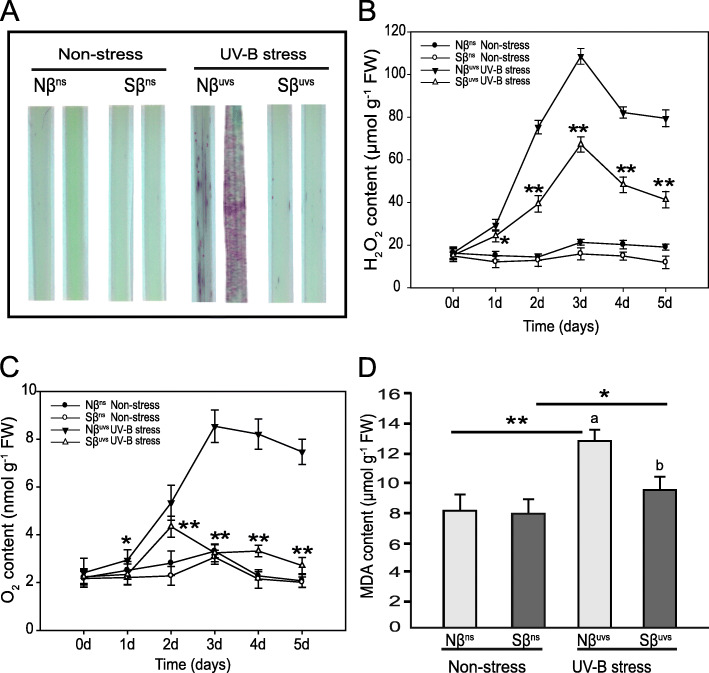
Differences in ROS accumulation of Nβ and Sβ plants under non-stress and stress conditions. **A** The Nβ and Sβ leaves were evaluated for superoxide anions using NBT staining under non-stress and UV-B stress; further, the contents of **B** H_2_O_2_ and **C** O_2_ were measured each day during the entire period of UV-B stress (1–5 days). Evaluation of lipid peroxidation was conducted by measuring **D** MDA levels. The means with different letters are significantly different (P < 0.05) within the same treatment. Lines with asterisks indicate significant differences between non-stress and UV-B stress conditions of the same plants (Nβ^ns^ vs. Nβ^uvs^ and Sβ^ns^ vs. Sβ^uvs^), as determined by Student’s t test (*P < 0.05, **P < 0.01)

One of the many consequences in UV-B-stressed plants is the accumulation of high levels of ROS in cellular compartments that has harmful effects on cell homeostasis, structures, and functions and results in oxidative stress. In order to understand the effect of βSito on cell membrane integrity and oxidative stability of rice plants under prolonged UV-B irradiation, we have initially analyzed the amount of H_2_O_2_ and O_2_ in Nβ and Sβ plants under non-stress and after UV-B stress continuously until 5 days of treatment. Interestingly, H_2_O_2_ and O_2_ contents in Nβ^uvs^ vs. Sβ^uvs^ plants were not different under non-stress conditions, but the levels of these ROS were markedly abundant in Nβ^uvs^ plants as compared to Sβ^uvs^ plants during the entire period of stress (Fig. 4B, C). Moreover, when the leaves were visualized using the NBT staining method, significantly intense accumulation of intracellular ROS was detected in Nβ^uvs^ plants than in Sβ^uvs^ plants (Fig. 4A). Further, to assess membrane damage by lipid peroxidation, MDA contents were measured in plants under non-stress and stress conditions. Although MDA level was slightly increased in Sβ^uvs^ vs. Sβ^ns^ plants, the magnitude of increase was much higher in Nβ^uvs^ plants (up to 12.78 μmol g^−1^ FW) that shows an elevated activity of lipid peroxidation in Nβ plants after stress (Fig. 4D).

**Fig. 5:**
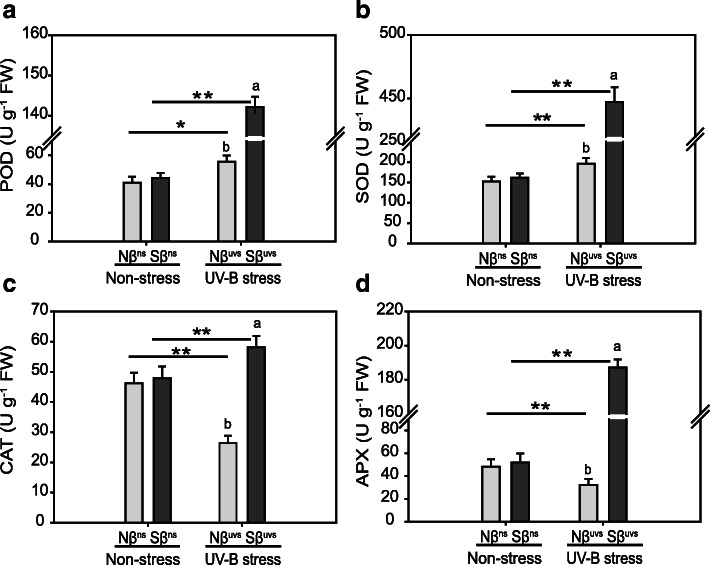
Activities of ROS scavenging enzymes in Nβ and Sβ plants under non-stress and UV-B stress conditions. We measured the activities of antioxidant enzymes, **A** peroxidase (POD), **B** superoxide dismutase (SOD), **C** catalase (CAT), and **D** ascorbate peroxidase (APX), in seedlings of Nβ and Sβ plants. The means with different letters are significantly different (P < 0.05) within the same treatment. Lines with asterisks indicate significant differences between non-stress and UV-B stress conditions of the same plants (Nβ^ns^ vs. Nβ^uvs^ and Sβ^ns^ vs. Sβ^uvs^), as determined by Student’s t test (*P < 0.05, **P < 0.01)

Furthermore, abundant evidence supports the idea that the equilibrium between the production and detoxification of ROS is sustained by antioxidant enzyme activities thereby maintaining ROS at low concentrations in stressed plants and thus allowing them to perform normal functions. Therefore, we measured the activities of antioxidant enzymes (POD, SOD, CAT, and APX) in Nβ and Sβ plants under non-stress and stress conditions. Under non-stress conditions, the difference in activities of antioxidant enzymes was not changed between Nβ^ns^ and Sβ^ns^ plants, but we recorded a dramatic increase in SOD (446.32 U g^−1^ FW), POD (143.21 U g^−1^ FW), CAT (58.44 U g^−1^ FW), and APX (184.57 U g^−1^ FW) activities in Sβ^uvs^ plants (Fig. 5). In case of Nβ^uvs^ plants, we detect only a slight increase in SOD and POD activities as compared to non-stress conditions, whereas CAT and APX activities (27.81 U g^−1^ FW and 33.42 U g^−1^ FW, respectively) were reduced after exposure to prolonged UV-B light (Fig. 5). These results demonstrate an elevated level of oxidative stress tolerance in Sβ plants under stress through avoiding oxidative damage caused by UV-B. Maintaining a high level of antioxidant enzymes in Sβ plants eventually helps plants to immediately scavenge ROS in cells and thus restore redox homeostasis.

### Effects of β-sitosterol on metabolic profile of rice plants during UV stress

**Fig. 6:**
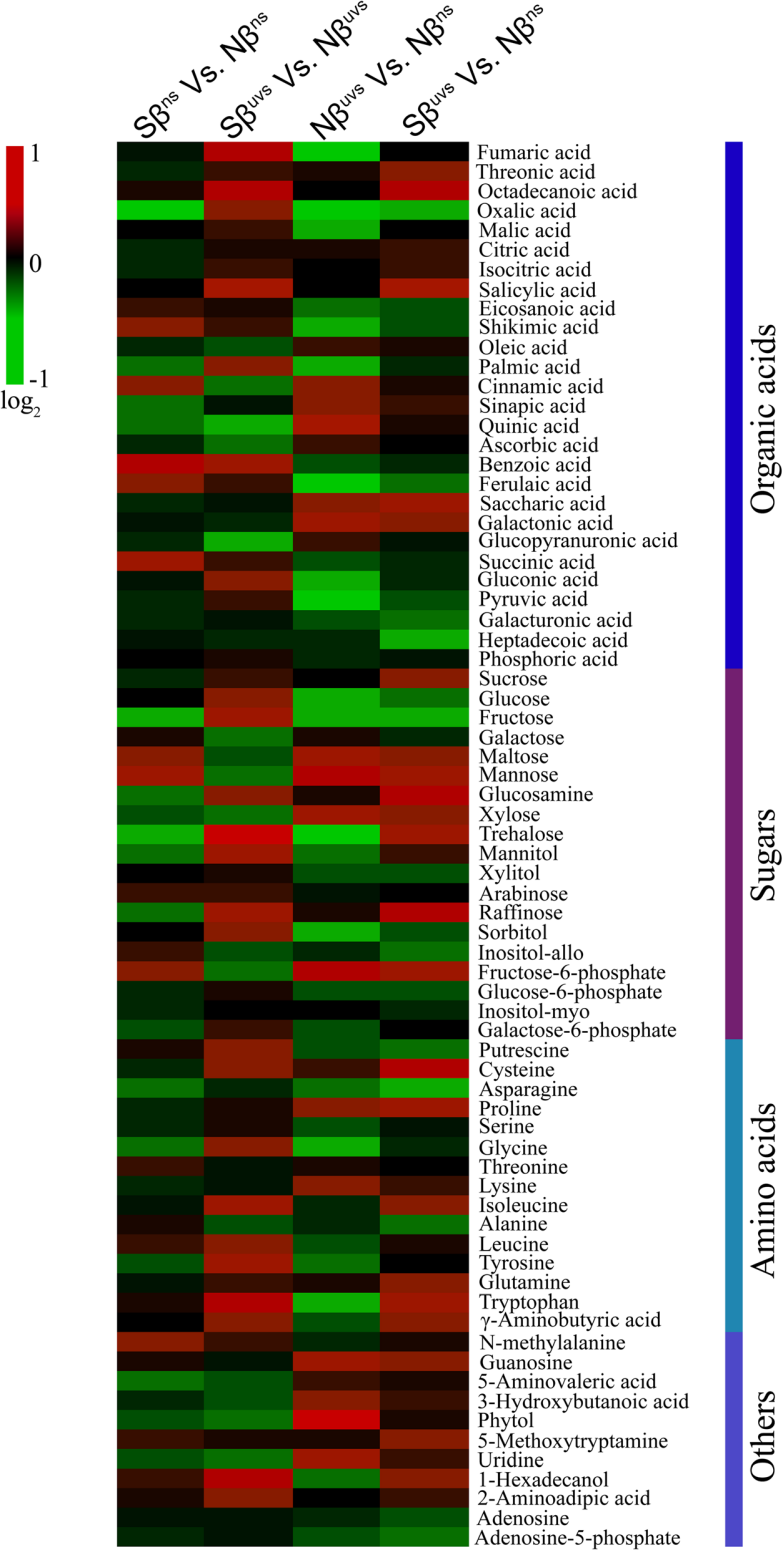
Heat map of 72 key metabolites in Nβ and Sβ plants under non-stress and UV-B stress conditions. The heat map shows the log_2_ fold change ratios, where red color codes representing upregulation whereas green color codes show downregulation of metabolites. The data is representative of two independent experiments

To investigate the effect of βSito on the biochemical snapshot of rice plants subjected to UV-B stress, we utilized a metabolomics approach to quantify the changes in specific metabolites in Nβ and Sβ plants under non-stress and after UV-B stress. A total of 72 key metabolites were detected and quantified using GC-TOFMS in leaf samples from Nβ and Sβ plants, out of which 27 belongs to organic acids, 19 belongs to sugars, 15 were amino acids, and 11 others (Supplementary Table S1). Differences in metabolite contents are visualized as a heat map that shows the overall changes in 72 metabolites under non-stress and UV-B stress conditions (Fig. 6). Under UV-B stress conditions, a large number of metabolites were induced in Sβ^uvs^ vs. Nβ^uvs^ plants, whereas a significant decrease in accumulation of several metabolites was recorded in Nβ^uvs^ vs. Nβ^ns^ plants (Fig. 6). However, Sβ^uvs^ vs. Nβ^uvs^, we have also noticed a reduction in levels of some organic acids and sugars. This implies a significantly different mechanism of metabolite regulation in Sβ and Nβ plants subjected to UV-B stress. Under non-stress conditions, we have also observed different metabolite accumulation patterns (Sβ^ns^ vs. Nβ^ns^), with the most obvious differences recorded for organic acids and sugars (Fig. 6). In case of Sβ^uvs^ vs. Nβ^ns^, sugars, amino acids, and other key metabolites were mainly induced, while no obvious differences were noted for organic acids (with regard to total organic acids) accumulation (Fig. 6). In total, these results thus demonstrate that exogenous application of βSito has significantly induced several metabolites belonging to organic acids, sugars, and amino acids as well some other key metabolites in Sβ plants compared with Nβ plants under stress. Contrastingly, we recorded a significant downregulation of several metabolites belonging to organic acids, sugars, and amino acids in Nβ plants under UV-B stress (Fig. 6). Importantly, these results also imply that application of βSito has mainly affected the accumulation of various metabolites in rice plants under UV-B stress (Sβ^uvs^ vs. Nβ^uvs^) rather than unstressed plants (Sβ^ns^ vs. Nβ^ns^).

### Effects of β-sitosterol on accumulation of key metabolites belonging to different metabolic pathways

**Fig. 7:**
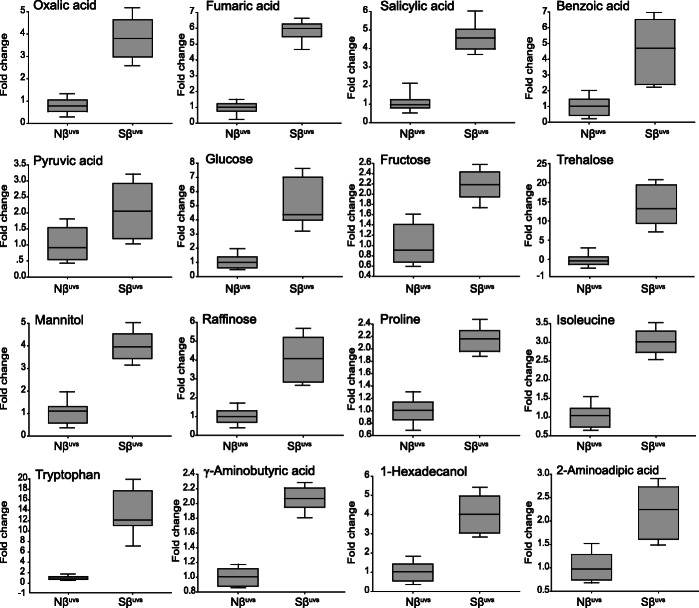
Metabolite abundance of selected key metabolites in Nβ and Sβ plants after UV-B stress. Box plots showing the relative abundance of key metabolites of sugar and amino acid metabolism, GABA shunt, and tricarboxylic acid (TCA) cycle, represented as fold change in Nβ and Sβ seedlings after stress

After prolonged UV-B stress, significantly reduced levels of few organic acids were observed especially fumaric acid, oxalic acid, malic acid, shikimic acid, palmic acid, ferulaic acid, and pyruvic acid in Nβ plants as compared to non-stress plants (Nβ^uvs^ vs. Nβ^ns^, Fig. 6). In contrast, several organic acids were accumulated in large amounts in Sβ^uvs^ vs. Nβ^uvs^ plants, such as 4.5-fold increase in oxalic acid, 6.2-foldsincrease in fumaric acid, 5-fold increase in salicylic acid, and 6.3-fold increase in benzoic acid followed by 2.6-fold increase in pyruvic acid (Figs. 6 and 7). In case of sugars, we found that glucose, fructose, sorbitol, and trehalose were reduced in Nβ^uvs^ vs. Nβ^ns^ plants (Fig. 6 and Supplementary Table S1). Analysis of sugars in Sβ^uvs^ vs. Nβ^uvs^ plants reveals that trehalose, mannitol, glucose, fructose, and raffinose were increased significantly (up to 19-folds, 4.5-folds, 6-folds, 2.3-folds, and 5.1-folds, respectively) after stress is applied (Figs. 6 and 7). Conversely, the levels of these sugars were low in Nβ^uvs^ vs. Nβ^ns^ plants (Fig. 6 and Supplementary Table S1). In case of amino acids, we found an obvious reduction in the levels of glycine and tryptophan in Nβ plants under UV-B stress as compared to non-stress (Nβ^uvs^ vs. Nβ^ns^, Fig. 6 and Supplementary Table S1). Interestingly, the relative abundance of amino acids such as tryptophan, isoleucine, and γ-aminobutyric acid (GABA) was increased up to 17-folds, 3.2-folds, and 2.2-folds, respectively, in Sβ^uvs^ vs. Nβ^uvs^ plants (Figs. 6 and 7). For proline, we noticed an increased accumulation in Nβ^uvs^ vs. Nβ^ns^ and Sβ^uvs^ vs. Nβ^ns^, but the level of increase was higher in Sβ^uvs^ vs. Nβ^ns^ plants (Fig. 6). Analyzing the other key metabolite profile in Sβ^uvs^ vs. Nβ^uvs^ plants, we found 4.9-folds and 2.7-fold increase in 1-hexadecanol and 2-aminoadipic acid, respectively (Fig. 7). Accumulation of different metabolites is crucial for the activation of various signaling and biochemical pathways in plants to overcome environmental challenges especially under high UV-B light. Differential accumulation of metabolites (including organic acids, sugars, amino acids, and others) between Nβ and Sβ plants suggest that various metabolic pathways are regulated in plants through β-sitosterol, which further explains the underlying mechanisms of UV-B stress tolerance in Sβ plants. Overall, these results imply that βSito is an essential part of stress signaling and participates in the tolerance mechanism against UV-B in rice. Importantly, differential accumulation of several key metabolites in Sβ plants is regulated via exogenous application of βSito and is associated with sugar and amino acid metabolism, GABA shunt, and tricarboxylic acid (TCA) cycle.

## Discussion

Over the last two decades, many studies have been conducted to understand the morpho-physiological and biochemical effects of UV-B light in plants and to reveal the mechanisms of resistance especially in UV-sensitive economically important higher plants. Rice is one of the most important crops in the world. Previous studies reported that different rice cultivars are sensitive to UV-B radiation although belonging to the same ecotype [33, 34]. In this study, we reported the positive impact of exogenous application of β-sitosterol (βSito) on rice plants subjected to prolonged UV-B irradiation. Our results demonstrate that βSito could significantly improve various aspects of growth and development when plants were subjected to UV-B stress and thus play a vital role in UV tolerance mechanisms in rice plants. The rice plants treated with βSito (Sβ plants) maintained their normal functions specifically photosynthesis-related processes that are generally sensitive to any stress conditions. Total chlorophyll and carotenoid levels were significantly higher in Sβ^uvs^ plants than in Nβ^uvs^ plants under UV-B stress (Fig. 2). Moreover, ultrastructure examination of chloroplast grana revealed abnormal grana stacks only in Nβ^uvs^ plants, which remain intact in Sβ^uvs^ plants as compared to non-stress conditions (Fig. 2). Further, the rate of net photosynthesis (Pn) was significantly higher with relatively constant WUEi in Sβ^uvs^ plants (Fig. 3), which clearly indicates the plasticity of Sβ plants and shows the important role of βSito as a plant growth regulator. An interesting report has recently shown that βSito could serve as an important growth regulator and play protective role under stress conditions [16]. Recently, Li et al. [13] reported that βSito treated plants had significantly higher Chl, Pn, and WUEi under stress conditions as compared to untreated plants. It is important to mention that the sterol biosynthetic pathway in plants is complex that begins with squalene and is followed by its conversion to various end products. The positive role of different forms of sterols in various abiotic stresses such as low temperature and drought stress has also been reported earlier [35, 36].

At the cellular level, UV-B radiation strongly promotes ROS levels (include H_2_O_2_, O_2_, and O_2_·−), which subsequently oxidizes proteins, lipids, and other biomolecules. This process of oxidation leads to abnormal functionality of cells by destabilizing the integrity of enzymes and cell membranes [7]. Our current study shows that the amount of ROS was increased in Nβ^uvs^ plants as demonstrated by elevated levels of H_2_O_2_ and O_2_ as well as superoxide anions (verified through NBT staining), whereas significantly lower levels of ROS were detected in Sβ^uvs^ plants (Fig. 4). Interestingly, we recorded low levels of MDA content in Sβ^uvs^ plants upon UV-B exposure but elevated levels in Nβ^uvs^ plants (Fig. 4). The measurement of MDA content has long been used as a lipid peroxidation marker in studies related to oxidative stress and redox signaling, and its presence in a low amount determines high tolerance in terms of decrease oxidative stress [37].

ROS scavenging mechanisms could be categorized mainly into two types in plants which include enzymatic and non-enzymatic defense systems. In the enzymatic defense system, the main players are antioxidant enzymes (SOD, POD, CAT, APX, etc.) which function together to detoxify ROS and thus maintain the redox homeostasis [8]. In the current study, we subsequently analyzed the activities of antioxidant enzymes, and the results show a dramatic increase of antioxidant enzyme activities in Sβ plants upon UV-B irradiation (Sβ^uvs^ vs. Nβ^uvs^ and Sβ^uvs^ vs. Sβ^ns^). However, we detected much lower activities of these enzymes in Nβ plants after stress application (SNβ^uvs^ vs. Sβ^uvs^, Fig. 5). This result clearly indicated that pretreatment of rice plants with βSito activates defense signaling only in Sβ plants via antioxidant enzymes against UV-B-induced ROS levels. Our results are in agreement with a previous study that shows a significant increment in activities of antioxidant enzymes under water stress conditions in wheat plants pretreated with βSito [16]. The same study also reported a significant increase in relative expression levels of SOD and dehydrin gene in plants pretreated with βSito which clearly reveals the βSito-regulated antioxidant system in plants under stress conditions. Importantly, improvement of oxidative status by βSito was also reported in animal systems that ultimately enhanced the growth performance and meat quality of animals [38].

Another widely recognized mechanism of plants to minimize stress-induced oxidative damage to cells and cellular components is the alteration in the levels of various metabolites which finally shape up different metabolic pathways [39, 40]. Since direct exposure to UV-B radiation could lower the antioxidant capacity of plants [41], consequently, metabolic alterations could compromise the plant defense system. In this study using the metabolomics approach by GC-TOFMS, we detected 72 key metabolites including organic acids, sugars, amino acids, and others in Nβ and Sβ plants under non-stress and after UV-B stress (Fig. 6, Supplementary Table S1). We found a significant accumulation of several key metabolites in Sβ^uvs^ vs. Nβ^uvs^ plants, the levels of which were suppressed in Nβ plants after stress is applied (Nβ^uvs^ vs. Nβ^ns^, Fig. 6). For organic acid metabolites in Sβ^uvs^ vs. Nβ^uvs^ plants, we have observed higher accumulation such as 4.5-fold increase in oxalic acid, 6.2-fold increase in fumaric acid, 5-fold increase in salicylic acid, and 6.3 fold increase in benzoic acid followed by 2.6-fold increase in pyruvic acid (Figs. 6 and 7). Previous studies show that organic acid metabolites are intermediates of major carbon metabolism events such as photosynthesis and respiration in plants [42]. Moreover, oxalic acid was reported to serve as a signaling molecule to elevate the defense level in cucumber plants [43]. Interestingly, previous evidence show that salicylic acid positively regulates oxidative stress through the activation of antioxidant enzymes [44]. In general, organic acids are essential for maintaining pH and redox balance in plants during the normal TCA cycle to avoid oxidative stress due to ROS [45].

UV-B stress can induce fluctuations in carbohydrate metabolism thereby affecting CO_2_ assimilation and partitioning of sugars from source to sink. Soluble sugars such as glucose, fructose, and sucrose accumulate more in the cytoplasm and play a crucial role as plant osmolytes during various stresses [46]. Sugars are also important to supply energy for plant growth [47]. In our study, analysis of sugars in Sβ^uvs^ vs. Nβ^uvs^ plants reveal that trehalose, mannitol, glucose, fructose, and raffinose were increased significantly (up to 19-folds, 4.5-folds, 6-folds, 2.3-folds, and 5.1-folds, respectively) after stress is applied (Figs. 6 and 7). Conversely, the levels of these sugars were low in Nβ^uvs^ plants when stress is applied (Nβ^uvs^ vs. Nβ^ns^, Fig. 6 and Supplementary Table S1). Trehalose is important for maintaining ionic dynamics in leaf and stem tissues during stress [48]. The amount of mannitol levels may increase in response to UV-B stress for an osmoprotective and antagonistic function against free radicals and thus stabilized the structure of macromolecules [19]. It is also notable that glycolysis which is an oxygen-independent metabolic pathway of respiration converts glucose into pyruvate and provides energy [49]. In the current study, the levels of glucose and pyruvic acid were significantly triggered in Sβ plants indicating βSito-regulated glycolysis which might be due to the upregulation of enzymes involved in the glycolytic pathway. In case of amino acids, low levels of several metabolites were recorded in Nβ^uvs^ vs. Nβ^ns^ plants after stress; however, accumulation of many amino acids was induced in Sβ^uvs^ vs. Nβ^uvs^ and in Sβ^uvs^ vs. Nβ^ns^ (Figs. 6 and 7). For proline, we noticed an increased accumulation in Nβ^uvs^ vs. Nβ^ns^ and Sβ^uvs^ vs. Nβ^ns^, but the level of increase was higher in Sβ^uvs^ vs. Nβ^ns^ plants (Fig. 6). For tryptophan, significantly low levels were detected in Nβ^uvs^ vs. Nβ^ns^ but increase in Sβ^uvs^ vs. Nβ^uvs^ and in Sβ^uvs^ vs. Nβ^ns^ (Figs. 6 and 7). Apparently, proline serves as stress-related signal and participates across different metabolic pathways including TCA and the phenylpropanoid pathways [50]. Tryptophan is another important amino acid which is required for the formation of various secondary growth metabolites, namely phytoalexins, indole glucosinolates, alkaloids, and serotonin. The spatial and temporal distribution of these metabolites is necessary for plant defense and acclimatization under stress conditions [51]. Generally, free amino acids are constituents of proteins, protein chaperons, and precursors for secondary metabolites and serve as signaling molecules to protect plants during interaction with the environment [52]. Further, the non-enzymatic mechanism of detoxification of free radicals in plants is regulated through alterations in secondary metabolites [53]. These secondary metabolites have broad-spectrum defensive roles, such as enzyme modulators and enzymatic activity, allelopathic, protection against UV-absorption, defense against herbivores, and antioxidant to neutralize free radicals [54]. Based on this evidence, it is highly likely that the application of βSito enhanced the tolerance level of Sβ plants through reprogramming of several core metabolites that eventually shape up the primary and secondary metabolism in rice plants under UV-B stress.

## Conclusions

In this study, we concluded that exogenous application of the β-sitosterol contributes positively to plant growth and stress signaling in rice plants during prolonged UV-B stress. The shoot length and root length was not affected in Sβ plants under UV-B stress (Sβ^uvs^); however, a sharp decline in the shoot (57.15% decrease in Nβ^uvs^ vs. Nβ^ns^, 64% decrease in Nβ^uvs^ vs. Sβ^uvs^) and root (40% decrease in Nβ^uvs^ vs. Nβ^ns^, 42% decrease in Nβ^uvs^ vs. Sβ^uvs^) length was recorded in Nβ^uvs^ plants. As a result, we found a substantial decrease of plant biomass in Nβ^uvs^ plants, i.e., nearly 52% lower as compared with Sβ^uvs^ plants under prolonged UV-B irradiation stress. The photosynthetic-associated characteristics were also measured which show a 27.78% increase of net photosynthetic rate (Pn) in Sβ^uvs^ plants (vs. non-stress plants). Other related parameters also show an increasing trend such as Gs, Fv/Fm, and NPQ in Sβ plants after stress is applied. In contrast, 52.20% and 26.67% reduction in Pn (Nβ^uvs^ vs. Sβ^uvs^ and Nβ^uvs^ vs. Nβ^ns^, respectively) was observed in Nβ^uvs^ plants. Consistently, photosynthetic pigments such as chlorophyll and carotenoids were higher in Sβ plants; however, pigment contents were significantly reduced in Nβ plants after stress. All these findings show a positive correlation of β-sitosterol application with growth- and development-related characteristics in rice plants during prolonged UV-B stress. Another important part of this research shows that Sβ plants exhibit more plasticity to UV-B irradiation due to stabilization of the antioxidant system in Sβ plants. Activities of antioxidant enzymes including SOD, POD, CAT, and APX were enhanced only in Sβ plants while decreased significantly in Nβ plants upon UV-B exposure. Further, a significant accumulation of several core metabolites that include organic acids, sugars, amino acids, and others in Sβ plants reveals βSito-regulated activation of non-enzymatic antioxidant mechanism in rice plants under stress. Overall, this study is beneficial especially in terms of its involvement in plant growth and development; moreover, βSito-regulated plasticity of plants during UV-B irradiation shows that its application could further be explored specifically in relation to different environmental stresses in other economically useful crop plants.

## Supplementary Information

**Additional file 1: Table S1.** Table showing different relative concentration of 72 metabolites (organic acids, amino acids, sugars, and others) in triplicate. ns - non-stress, uvs - UV-B stress.

## Data Availability

All the data is within this manuscript.

## Abbreviations

PPFD: Photosynthetic photon flux density
RWC: Relative water content
GABA: γ-Aminobutyric acid
TCA: Tricarboxylic acid cycle
MDA: Malondialdehyde
NPQ: Non-photochemical quenching
DMSO: Dimethyl sulfoxide
βSito: β-Sitosterol (chemical compound)
ROS: Reactive oxygen species
WUEi: Water use efficiency (intrinsic)
CAT: Catalase
SOD: Superoxide dismutase
POD: Peroxidase
APX: Ascorbate peroxidase
